# Translating investments into results: the role of the World Bank in global health

**DOI:** 10.1136/bmjgh-2025-019078

**Published:** 2025-10-23

**Authors:** Kiran Raj Pandey, Bipin Adhikari, Lorenz von Seidlein

**Affiliations:** 1 Medharma Clinics, Kathmandu, Bagmati, Nepal; 2 Nuffield Department of Medicine, University of Oxford, Oxford, UK; 3 Mahidol University, Salaya, Thailand; 4 University of Oxford, Oxford, UK

**Keywords:** Global Health, Health systems, Health economics

**Summary box**

- Over the past few decades, the World Bank Group has emerged as an important stakeholder in global health, following decades of its investments in health systems around the world.
- This commentary highlights that the recently concluded replenishment round of the World Bank Group’s International Development Association will mobilise approximately $100 billion in some of the world’s poorest countries, offering significant opportunities to invest in health services and systems in these countries.
- This commentary calls for the World Bank Group to actively engage with the global health community in deciding how its investments in health services and systems are used to effectively translate these financial resources into improved health outcomes.

In December 2024, the International Development Association (IDA)—the World Bank Group’s concessional lending arm—concluded its 21st replenishment round. IDA21, as this replenishment round is referred to in World Bank Group nomenclature, was substantial with donors committing a record 24 billion dollars for a 3-year period.^1^

The World Bank Group leadership welcomes this replenishment as a record haul. The optimism may be justified but could be short-lived if the IDA is not thoughtful about how that money is going to be spent. The global health community has reasons to be hopeful, but it also needs to be watchful.

The 24 billion dollar commitment will allow the IDA to invest about $100 billion—buttressed by leverage and other financial instruments—in 78 of the world’s poorer economies over the next 3 years.^1^ This is an increase from the $93 billion earmarked for the previous replenishment cycle covering years from 2022 to 2025. A sizeable part of this $100 billion investment will be made in health services and systems, under what the IDA calls its ‘people’ focus area.^2^

The World Bank Group—through its constituent entities the IDA, the International Bank for Reconstruction and Development (IBRD) and the International Finance Corporation—is now a prominent stakeholder in global health, offering not just investment but also technical cooperation in health services, systems and policy in much of the developing world. While the IBRD, the Group’s main lending arm, lends mostly to more economically mature countries, the IDA provides grants and concessional loans with long maturities to countries that otherwise would not be able to meet their financing needs for infrastructure, health, education and similar areas.

Over the last 30 years, the IDA and the IBRD have increasingly shifted their attention and investments towards health and social services spending. The Group’s Health Nutrition and Population (HNP) practice has seen a substantial increase in the investment it has made across the world. Between 1989 and 1993, the IDA and the IBRD’s total commitment to its HNP practice averaged at $480 million per year (in current US$). By 1999, that figure had increased three-fold to $1·5 billion per year (figure 1).^3^

**Figure 1:**
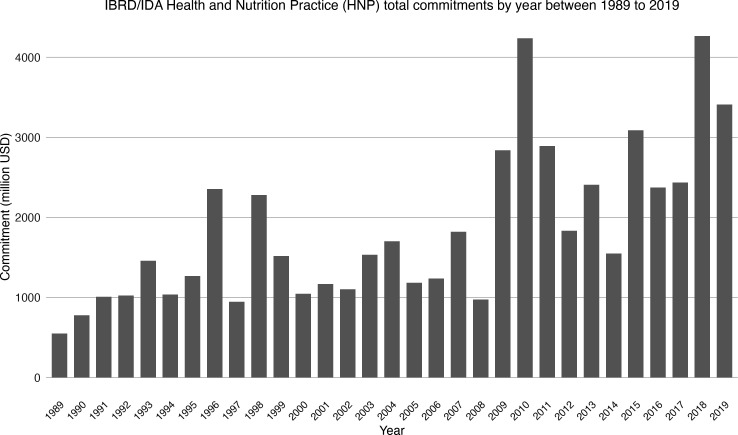
Annual World Bank commitments to the Health, Nutrition and Population (HNP) sector through IBRD and IDA financing, 1989–2019. Data represent total yearly commitments in million US$ (in current US$). Data sourced from the World Bank data portal at https://datatopics.worldbank.org/health/lending. IBRD, International Bank for Reconstruction and Development; IDA, International Development Association.

The World Bank Group’s growing health portfolio now exceeds $27 billion invested in over 160 projects across the world. And its ambitions do not end there. By 2030, the Group’s HNP practice aims to ‘support countries in delivering quality, affordable health services to 1·5 billion people’.^4^ This broad commitment of resources and attention to the health of the world’s neediest people from the world’s largest multilateral financial institution raises hope, especially because the World Bank did not prioritise investing in health before.

The pivotal change in priorities was established in 1993 when the World Bank’s flagship publication, the annual World Development Report, made a case for investing in the health of the world’s poor people. That report, aptly called ‘Investing in Health’, argued that public as well as private investing in health would lead to substantial gains for society and encouraged governments as well as private entities to invest in health systems and services.^5^

The World Bank Group backed its policy recommendations to national governments by investing its own money in health. Between 1997 and 2007, the World Bank invested $15 billion in health, nutrition and population activities. However, by the Bank’s own admission, the investment and efforts did not produce the desired impact. A new strategy directive for its HNP practice, released in 2007, tried to make HNP programming more results-oriented.^6^ Directed by its 1997 strategy document, the Bank’s investment priorities in health changed from family planning and nutrition to efforts at improving health systems performance and securing sustainable health financing.^7^ Although the Bank’s investment in health has accelerated since then—in 2019 alone, the IDA and IBRD invested $3·4 billion in health—showing results has not been easy.

Following its decision to increase investments in health, the World Bank Group also made technical recommendations to member countries on increasing investments in health and improving the cost-effectiveness of investments. Inadequate financing was identified as critically important for health services and systems improvement and policy interventions such as health insurance were thought to help achieve this policy objective.^8^

Demand-generating interventions such as health insurance were also expected to help achieve another policy objective of promoting provider pluralism and market-based approaches in health services provision. These interventions were also expected to improve consumer choice through competition; reduce financial hardship due to the use of health services; and improve provider accountability, health services and systems.^9 10^ The World Development Report 2004, expectantly titled ‘Making services work for poor people’, stated ‘…services can be improved by putting poor people at the centre of service provision. How? By enabling the poor to monitor and discipline service providers, by amplifying their voice in policy making, and by strengthening the incentives for providers to serve the poor.’^9^

Health financing has gradually improved in many developing countries since then,^11^ but has failed to address supply-side challenges, including the type, quality and availability of health services. For instance, India’s national health insurance scheme introduced in 2007 aimed to reduce demand-side barriers and financial hardship by offering financial support for hospital care. This insurance scheme, then called Rashtriya Swasthya Bima Yojana, allowed households to access inpatient services at public as well as private hospitals, by paying a nominal annual premium. In Nepal, a similar health insurance scheme was launched that mostly covered inpatient services, alongside emergency and outpatient treatment.^12^

Evaluations of India’s insurance scheme have failed to show evidence of improved health outcomes while documenting an increase in financial hardship for beneficiaries of the insurance scheme, demonstrating a divergence between intended policy objectives and actual outcomes.^13 14^ Similar policy-outcome misalignment has not only been observed in India; the evidence on improvement in primary health outcomes after the implementation of health insurance in low and middle-income countries remains meagre at best.^15^,^18^

In the last decade, new evidence has shown that policy interventions that merely increase the financing for health services will not improve health outcomes.^14 19^ Where health outcomes have been shown to improve after the implementation of health insurance, it has been because insurance policies made careful considerations of supply side constraints such as the coverage offered, quality of care and provider incentives.^20 21^

To translate the World Bank Group’s investments in health into results and to make the greatest impact in health outcomes, there must be a careful consideration of not just how much money is spent on health, but also how that money is spent. To do this, the Bank must consider a few interrelated issues. The first issue relates to the quality of health services. By its own admission, the Bank is committed to ‘providing quality health services to everyone’.^4^ But despite the Bank’s efforts to make quality a priority in its funding efforts, gaps persist.

For example, an analysis of the Project Appraisal Documents for the World Bank hosted Global Financing Facility found significant gaps in addressing and operationalising quality of health services.^22^ What constitutes ‘quality health services’ and how to make them more accessible and affordable is a problem that beneficiaries, as well as health workers including doctors, nurses and public health workers, are uniquely situated to answer. People in the front lines of healthcare can contribute valuable insights—on how to effectively deliver high quality health services, align provider incentives to health systems goals and maximise improvement in health outcomes. They have a vital role to play in improving the quality of health services, and translating health investments into results. Without the insights and the active engagement of people in the frontlines of healthcare, it is unlikely that the Bank’s health investments will yield meaningful results.

This leads to the second issue—the power dynamics involved in the Bank’s policy processes and investment decisions. The power dynamics in these processes are such that they have failed to sufficiently engage frontline health workers, beneficiaries and other key stakeholders in the process.^23^ The Bank needs to engage proactively with the front line health workers, as well as beneficiaries in its policy process and investment decisions.

The third interrelated issue concerns the growing debt burden in developing countries that is squeezing out health investments in these countries. Between 2010 and 2023, developing countries’ foreign debt rose from 16% to 30% of the global debt burden. 3.3 billion people now live in countries that spend more on interest payments than on health.^24^ This situation raises strains over resources available for health services and systems in developing countries. Countries such as Kenya, Uganda and Nigeria are already facing popular protests as a consequence of mounting debt obligations squeezing out essential health services.^25^ Without a debt restructuring plan that goes alongside an investment plan, these countries will be left with a choice between forgoing essential health services or defaulting on their loan repayments. Were such a situation to transpire, decades of the World Bank Group’s investments in health would run the risk of being undone.

The World Bank Group’s investments in health are easily one of the biggest investments in health systems and services for the benefit of the world’s neediest people. Translating these investments into meaningful results is an important global concern, requiring thoughtful and collaborative global action. If the global health community wants to realise ambitious global goals,^26^ it cannot afford to stay disengaged from these investment decisions. And the World Bank Group, arguably the most powerful global health actor in terms of the investments it makes, needs to seek out, engage with and work alongside the global health community—including frontline health workers and beneficiaries—to realise the shared goal of a world where no person is left wanting of life-saving and life-affirming healthcare.

## Data Availability

All data relevant to the study are included in the article.
